# *Orthosiphon stamineus* protects *Caenorhabditis elegans* against *Staphylococcus aureus* infection through immunomodulation

**DOI:** 10.1242/bio.20148334

**Published:** 2014-06-27

**Authors:** Cin Kong, Man-Wah Tan, Sheila Nathan

**Affiliations:** 1 School of Biosciences and Biotechnology, Faculty of Science and Technology, Universiti Kebangsaan Malaysia, Bangi, 43600 UKM Bangi, Selangor, Malaysia; 2 Department of Genetics, Stanford University School of Medicine, Stanford, CA 94305-5120, USA; 3 Department of Microbiology and Immunology, Stanford University School of Medicine, Stanford, CA 94305-5124, USA; * Present address: Department of Infectious Disease, Genentech, Inc., South San Francisco, CA 94080, USA.

**Keywords:** *C. elegans*, *S. aureus*, *O. stamineus*, Anti-infective, Immunomodulation

## Abstract

Amidst growing concerns over the spread of antibiotic-resistant *Staphylococcus aureus* strains, the identification of alternative therapeutic molecules has become paramount. Previously, we utilized a *Caenorhabditis elegans*–*S. aureus* screening platform to identify potential anti-infective agents from a collection of natural extracts and synthetic compounds. One of the hits obtained from the screen was the aqueous extract of *Orthosiphon stamineus* leaves (UE-12) that enhanced the survival of infected nematodes without interfering with bacterial growth. In this study, we used a fluorescent transgenic reporter strain and observed that the repressed expression of the *lys-7* defense gene in infected nematodes was restored in the presence of UE-12. Analysis of a selected panel of PMK-1 and DAF-16-regulated transcripts and loss-of-function mutants in these pathways indicates that the protective role of UE-12 is mediated via the p38 MAP kinase and insulin-like signaling pathways. Further analysis of a panel of known bioactive compounds of UE-12 proposed eupatorin (C_18_H_16_O_7_) as the possible candidate active molecule contributing to the anti-infective property of UE-12. Taken together, these findings strongly suggest that the *O. stamineus* leaf extract is a promising anti-infective agent that confers an advantage in survival against *S. aureus* infection by modulating the immune response of the infected host.

## INTRODUCTION

*Staphylococcus aureus* is a leading human pathogen associated with high mortality and morbidity in a wide spectrum of hospital- and community-acquired infections (Klevens et al., 2007; Grundmann et al., 2010). Wound infections, severe skin infections, sepsis and metastatic infections of many organ systems make up typical infections caused by this pathogen (Archer, 1998). Since the introduction of penicillin, the deployment of virtually all antibiotics has been followed by the evolution of clinically significant antibiotic resistance (Perez et al., 2008). This phenomenon begs for fresh approaches to identifying anti-infectives with novel modes of action other than targeting pathogen viability to quell bacterial resistance.

Recently, the genetically tractable nematode *Caenorhabditis elegans* has been used extensively in the modeling of infectious diseases (Sifri et al., 2005). Although *C. elegans* has no adaptive immune system, it has comprehensive systemic immunity. There is growing appreciation that the nematode can serve as a powerful tool in drug discovery including identification of antifungal and antimicrobial compounds (Moy et al., 2006; Breger et al., 2007). To date, a wide and still expanding range of pathogens have been reported to infect *C. elegans* including the human pathogen *S. aureus* (Sifri et al., 2003; Wu et al., 2010; JebaMercy et al., 2011). *S. aureus* kills *C. elegans* via accumulation of large numbers of live bacteria within the intestinal tract (Sifri et al., 2003; Irazoqui et al., 2010) and several virulence determinants known to be important in mammalian pathogenesis are also required for full pathogenicity against nematodes (Sifri et al., 2003).

Previously, we successfully established a liquid-based *C. elegans*–*S. aureus* anti-infective screen platform that identified not only substances with anti-bacterial properties, but also hits that did not interfere with bacterial viability (Kong et al., 2014). We screened a number of natural extracts and synthetic compounds for anti-infective properties using this *C. elegans* system and identified two promising hits that protected the worms from both methicillin-susceptible *S. aureus* (MSSA) and methicillin-resistant *S. aureus* (MRSA) infection. Both hits did not affect bacterial replication *in vitro*; on the other hand, both were able to cause a significant reduction in *in vivo* intestinal bacterial loads (Kong et al., 2014). We therefore postulated that these extracts may act distinctly from conventional antibiotics by modulating or enhancing the host immune system to eradicate the pathogen.

In the present study, we extend the use of this *C. elegans*–*S. aureus* infection model to dissect the possible underlying mechanism of one of the promising hits obtained i.e. the local plant product, *Orthosiphon stamineus* leaf extract (henceforth referred to as UE-12), in protecting the host from infection. *O. stamineus* has been used as folk medicine for centuries in Southeast Asia to treat urinary tract infections, diabetes, hypertension and rheumatoid disease (Ameer et al., 2012). In Europe and Japan, the leaves of *O. stamineus* are consumed as tea, most commonly known as java tea. As the leaves are non-toxic to humans, the potential of UE-12 in enhancing the host immune system makes this extract an attractive target for the development of a new anti-infective. Through the use of transgenic GFP reporter worms, loss-of-function *C. elegans* mutants and transcriptome analysis, we unlock the host molecular mechanisms and pathways associated with the activity of UE-12. We also gain insight into the active constituent of UE-12 that contributes to its anti-infective property.

## RESULTS

### UE-12 restores the repressed expression of the *lys-7* defense gene

We had previously identified 14 natural extracts and 14 synthetic compounds that enhanced the survival by at least 2.8-fold relative to the untreated infected control worms (Kong et al., 2014). Of these 28 hits, 7 extracts and 13 compounds rescued the worms from infection by inhibiting *S. aureus* replication whilst another 7 extracts and 1 compound did not interfere with bacterial growth. We investigated 5 of the 8 hits that did not interfere with bacterial growth, which we term anti-infective candidates. We hypothesized that these anti-infective candidates protected the host from infection, not by direct bacteriostatic or bactericidal effects towards the bacteria, but, act on the host defense system via modulating or stimulating the immune response towards infection.

**Fig. 1.**
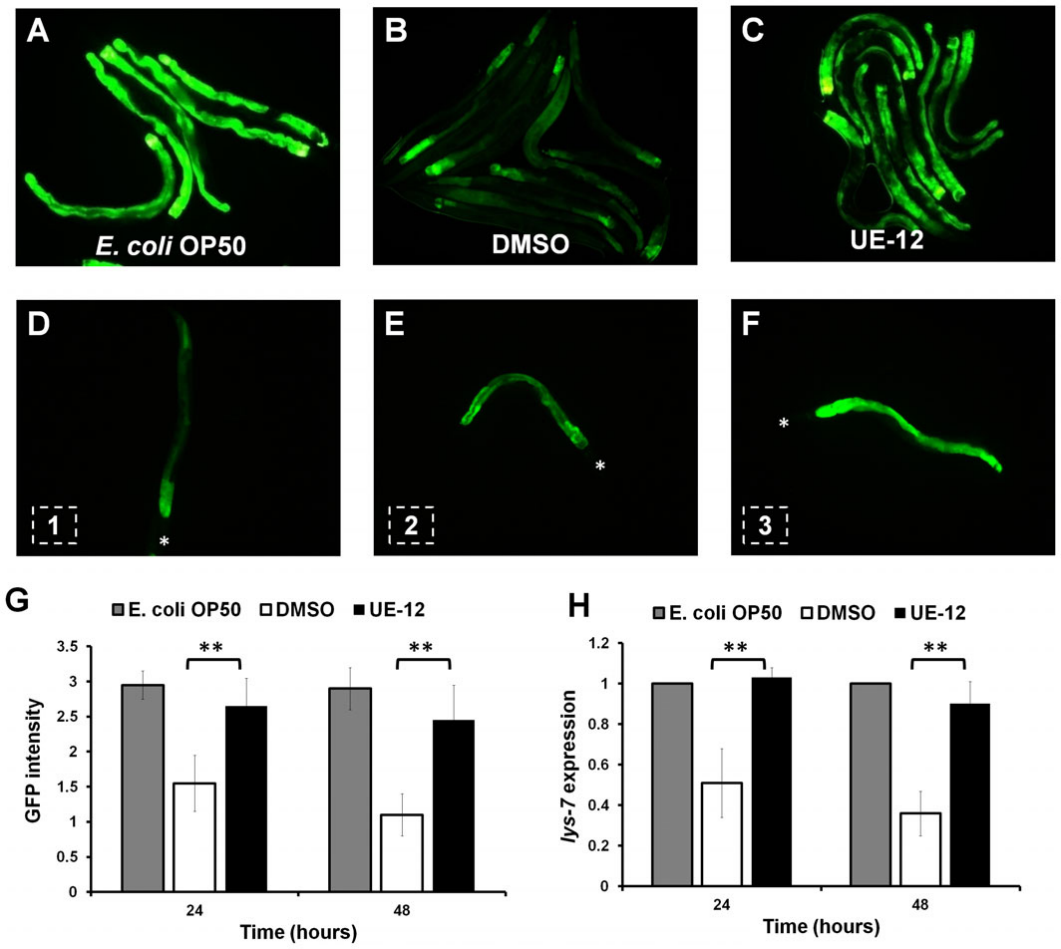
UE-12 replenishes the repressed expression of the *lys-7* defense gene. Representative fluorescence micrographs of the population of transgenic p*lys-7::gfp* worms (A) fed on *E. coli* OP50 (uninfected), (B) infected with *S. aureus* in the absence of UE-12 (animals were exposed to DMSO only) and (C) infected with *S. aureus* in the presence of UE-12 (100× magnification). (D,E,F) The criteria used for scoring of p*lys-7::gfp* transgene expression upon *S. aureus* infection in the presence and absence of extract. Each worm was given a score of 1, 2 or 3 depending on the GFP intensity observed. Asterisks mark the head of the worms. (G) Bars represent the mean ± SD of GFP intensity scores (expressed in arbitrary units) corresponding to the *lys-7* expression for each worm (n = 20) according to the criteria presented in panels D, E and F. (H) qRT-PCR analysis of *lys-7* mRNA shows significant induction of *lys-7* expression in infected animals following treatment with UE-12 at 24 and 48 hpi. **A significant difference between untreated (DMSO) and UE-12-treated worms (*p*<0.001).

To investigate how the anti-infective candidates affected the *C. elegans* antimicrobial response, we tested the effect of these candidates on the expression of a host immune effector using transgenic worms carrying a transcriptional GFP reporter for the *lys-7* gene. LYS-7 is an enzyme homolog of the antimicrobial lysozyme encoded by the *lys4* gene of the amoeboid protozoon *Entamoeba histolytica* (Leippe, 1999). The transgenic animals were infected by *S. aureus* in the presence and absence of anti-infective candidates and fluorescence intensity was visualized and compared at 24 and 48 hours post-infection (hpi). As shown in Fig. 1A and Fig. 1B, at 48 hpi, nematodes infected by *S. aureus* in the absence of anti-infective candidates showed a significant reduction in the overall GFP intensity as compared to the uninfected population fed on the normal food source, *Escherichia coli* OP50. The uninfected worms showed a consistently strong green fluorescence along the intestine throughout the observation period (Fig. 1A) whereas *S. aureus*-infected worms exhibited weak fluorescence except at the posterior part of the intestine (Fig. 1B). Interestingly, among the anti-infective candidates tested, the UE-12 extract was able to prevent suppression of GFP expression by *S. aureus* (Fig. 1C). By contrast, UE-01-4, UE-03-6, UE-11 and UC-10 failed to prevent the suppression of GFP expression by *S. aureus* (supplementary material Fig. S1). Next, we quantified the effect of UE-12 treatment on GFP intensity at 100× magnification. Worms were given a score of 1 when only the anterior part of the intestine fluoresced (Fig. 1D), a score of 2 for worms with intermediate fluorescence along the intestine (Fig. 1E) and a score of 3 for worms with intense fluorescence throughout the intestinal tract at 100× magnification (Fig. 1F). Based on the defined criteria, we confirmed that the green fluorescence intensity corresponding to *lys-7* expression was significantly higher in the infected worms treated with UE-12 compared to the non-treated infected worms (Fig. 1G, *p*<0.001, n = 20 worms per treatment).

We also quantified the mRNA levels of *lys-7* by quantitative real time PCR under the same experimental conditions. Consistent with the GFP data, UE-12 was able to prevent the suppression of *lys-7* expression by *S. aureus* at both 24 and 48 hpi (Fig. 1H, *p*<0.001). We thus selected UE-12 as our candidate anti-infective agent to elucidate its potential impact on host immunity.

### UE-12 confers a survival advantage against *S. aureus* infection by reducing the number of viable *S. aureus* in the *C. elegans* intestine

**Fig. 2.**
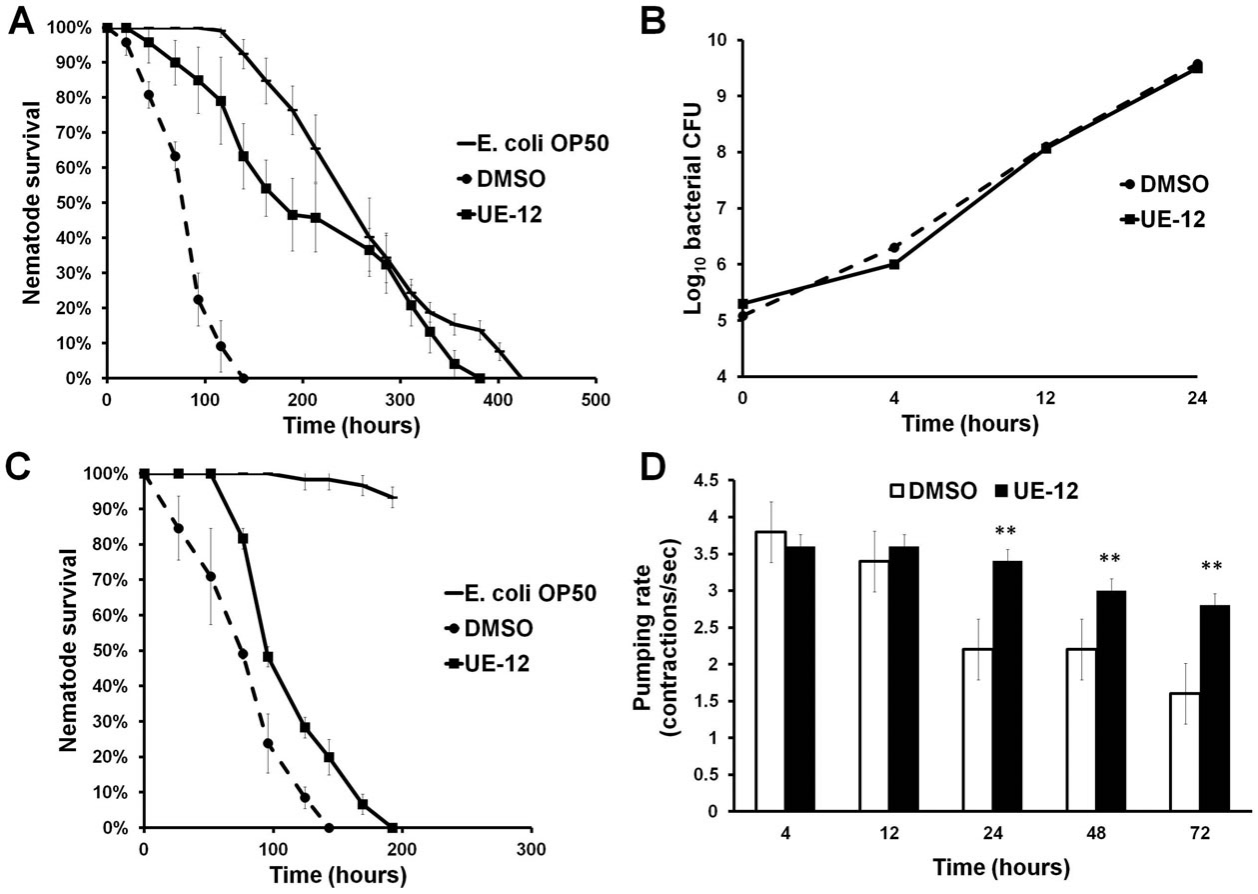
UE-12 enhances the survival of *S. aureus*-infected nematodes without interfering with bacterial growth. (A) *S. aureus* infection assay on wild-type N2 nematodes exposed to 200 µg/mL of UE-12 compared to untreated control exposed to solvent (DMSO) only. UE-12 confers significant lifespan extension in infected nematodes (*p*<0.0001). Graph shows the mean ± SD of six replicates (20 nematodes/replicate) from a representative of three independent assays. (B) UE-12 does not inhibit the growth of *S. aureus* in the same medium used for *C. elegans* infection assay, comprising 80% M9 buffer and 20% *S. aureus* culture. Data are the average of three independent experiments with error bars representing the standard error of the mean. (C) *S. aureus* infection assay on wild-type N2 nematodes pre-treated with 200 µg/mL of UE-12 for 24 hours compared to untreated control exposed to solvent (DMSO) only. The worms pre-treated with UE-12 were then infected with *S. aureus* in the absence of extract. The survival curves indicate significantly enhanced survival over time in UE-12-treated worms as compared to the control (*p*<0.0001). Graph shows the mean ± SD of six replicates (20 nematodes/replicate) from a representative of three independent assays. (D) No significant difference in pharyngeal pumping rates is observed for the first 4 and 12 hours. The pumping rates of untreated animals are significantly lower than the UE-12-treated animals after 24, 48 and 72 hours infection. The bars correspond to mean ± SD of the contractions/second from one representative of two individual replicates. **A significant difference between untreated (DMSO) and treated worms (*p*<0.001).

To further evaluate the protective effect of UE-12, we monitored the survival of both UE-12 and DMSO (control)-treated nematodes infected with *S. aureus* every 24 hours. An additional control group of worms exposed to *E. coli* OP50 was assayed in parallel. As expected, *S. aureus* killed the worms significantly faster with a mean time to death (TD_mean_) of 85.4±2.8 hours compared to the *E. coli* exposed population (270.9±7.6 hours; *p*<0.0001). However, in the presence of UE-12, *S. aureus*-infected worms had an overall survival that was significantly improved with a TD_mean_ of 215.8±9.4 hours (*p*<0.0001) (Fig. 2A). When the infection was performed using the conventional agar-based *C. elegans* infection assays, UE-12 was also able to enhance the survival of infected nematodes on the agar medium, albeit to a lesser extent compared to the liquid medium (supplementary material Fig. S2). This difference may be due to inadequate exposure or absorption of UE-12 extract by the host (Zheng et al., 2013). Given that the protection effect was more pronounced in the liquid-based assay, we chose this medium for all the following experiments.

We previously showed by disc diffusion and MIC microdilution tests that UE-12 at 2000 µg/ml did not inhibit or limit *S. aureus* growth *in vitro* (Kong et al., 2014). To further verify these findings, we examined the growth of *S. aureus* in the presence (200 µg/ml) and absence of UE-12 based on the number of CFU enumerated under similar conditions as the infection assay. The initial bacterial inoculum was ∼1.5×10^5^ and over time, an increase in the CFU indicated bacterial replication in medium consisting of 20% TSB and 80% M9 buffer confirming that UE-12 did not affect *S. aureus* growth (Fig. 2B). Hence, we have verified that the enhanced survival of infected worms in the presence of UE-12 extract was not due to the direct killing of *S. aureus* by UE-12.

To determine if the protective effect conferred by UE-12 is mediated via the host, we pre-treated the worms with UE-12 for 24 hours followed by washing in M9 medium and finally infecting these worms with *S. aureus* in the absence of UE-12. As shown in Fig. 2C, a significant lifespan extension was also observed in the infected worms pre-treated with UE-12 extract (*p*<0.0001). The TD_mean_ for UE-12-treated animals was significantly longer (118±4.6 hours) compared to the untreated infected population (83.2±4.6 hours). Nevertheless, the protective effect under these conditions was not as prominent as that observed when worms were continuously exposed to the extract (Fig. 2A), suggesting that continuous exposure to UE-12 is required to achieve the maximal anti-infective effect. This observed difference also proposes that UE-12 may target both pathogen virulence in tandem with host immunity.

In the earlier study, we further demonstrated a marked reduction in bacterial loads and no evidence of colonization in the gut of UE-12-treated infected nematodes at 12 and 24 hpi (Kong et al., 2014). To rule out the possibility that the lower intestinal burden was due to UE-12 decreasing pharyngeal pumping rates and thus, lowering uptake of bacteria, we determined pharyngeal pumping rates of *S. aureus*-infected animals in the presence of UE-12. As had been previously reported (Sifri et al., 2003; JebaMercy et al., 2011), as the infection continued, there was a time-dependent decline in pumping rates of untreated infected animals (Fig. 2D). Specifically, foraging activities and pharyngeal pumping in *S. aureus*-infected worms appeared to be normal for the first 16 to 20 hours, but progressively decreased after 24 and 48 hpi. UE-12-treated infected worms continued to feed normally at 4 and 12 hours following infection and continued to maintain significantly higher pumping rates at 24, 48 and 72 hours compared to untreated worms (*p*<0.001) (Fig. 2D). Thus, the low number of intestinal bacteria could not be attributed to a decrease in bacteria uptake. Instead, other *in vivo* factor(s) may be responsible for the suppression in bacterial loads within the host intestine.

### UE-12 does not affect the basal lifespan and reproduction of the worms

**Fig. 3.**
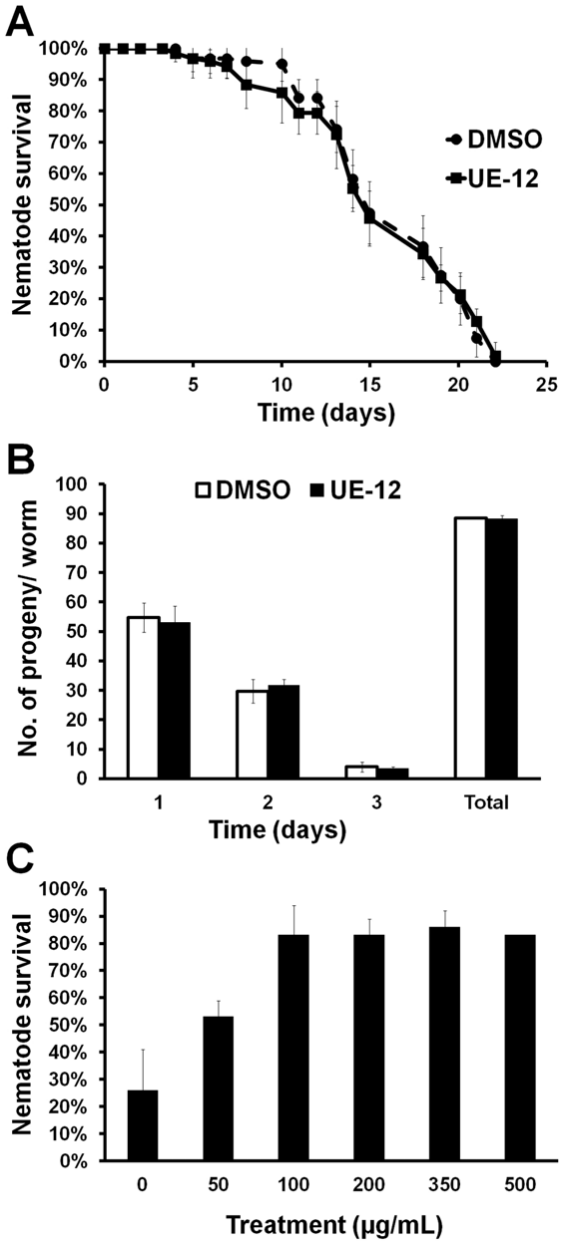
UE-12 does not affect the basal lifespan and reproduction of the worms. (A) UE-12 does not prolong or shorten the basal lifespan of uninfected worms. The graph represents the mean ± SD of six replicates (20 nematodes/replicate) from a representative of three independent assays. (B) Daily and cumulative reproduction output was determined from 10 individual worms. UE-12 does not influence reproduction in *C. elegans*. The error bars represent the standard error of the mean. (C) *S. aureus* infection assay of wild-type nematodes exposed to different concentrations of UE-12. Enhanced survival is observed in UE-12 at all concentrations tested (50, 100, 200, 350 and 500 µg/mL) in a dose dependent manner at 96 hpi. The bars show the mean ± SD for percentage nematode survival from one representative of two individual replicates.

Next, we asked if UE-12 is able to extend the basal lifespan of the worm leading to better survival during an infection by comparing the lifespan of uninfected *C. elegans* in the presence and absence of UE-12. Treatment with 200 µg/mL UE-12 was begun at the young adult stage in the presence of heat-killed *E. coli* OP50 to exclude any possible effect of UE-12 on the bacteria. Both treated and untreated animals had a mean lifespan of 22–23 days at 25°C in liquid medium (*p*>0.01) (Fig. 3A), indicating that the enhanced survival of *S. aureus*-infected worms exposed to UE-12 is unlikely to be a simple consequence of any lifespan extending property of UE-12. No shortening of lifespan was noted in UE-12-treated animals, indicating that UE-12 at this concentration (200 µg/mL) did not exert any toxic effect on the adult worms.

We also examined if treatment with UE-12 affected *C. elegans* reproduction by measuring egg-laying and progeny production of UE-12-treated animals. We found that the daily and total reproductive output of the worms did not differ significantly between UE-12-treated and untreated animals (*p*>0.01) (Fig. 3B). In addition, we did not observe any abnormality in the fecundity of the eggs and development of the progeny into different larva stages. This suggests that UE-12 did not affect the reproduction ability of the worms nor was there any toxic effect on the progeny.

Finally, we showed that UE-12 promoted the survival of *S. aureus*-infected nematodes in a dose-dependent manner maxing out at 100 µg/mL (Fig. 3C). The continued positive treatment effects at 500 µg/mL suggest that even at this concentration, UE-12 is not toxic to the worms.

### UE-12 promotes worm survival via the p38 MAP kinase and insulin-like signaling pathways

Previous studies have shown that three major highly conserved signal transduction pathways, the *nsy-1/sek-1/pmk-1* p38 MAP kinase pathway, the *daf-2/daf-16* insulin/IGF1 pathway and the *dbl-1*/TGF-β signaling pathway, play significant roles in the survival of the host during infection (Kim et al., 2002; Mallo et al., 2002; Garsin et al., 2003). Using readily available *C. elegans* mutants for these three major signaling pathways, we asked if any of these pathways mediate the protective effect of UE-12 against *S. aureus* infection. We hypothesized that if a specific pathway is required for the protective effect of UE-12, then UE-12 will be unable to promote survival of the associated mutant following infection.

**Fig. 4.**
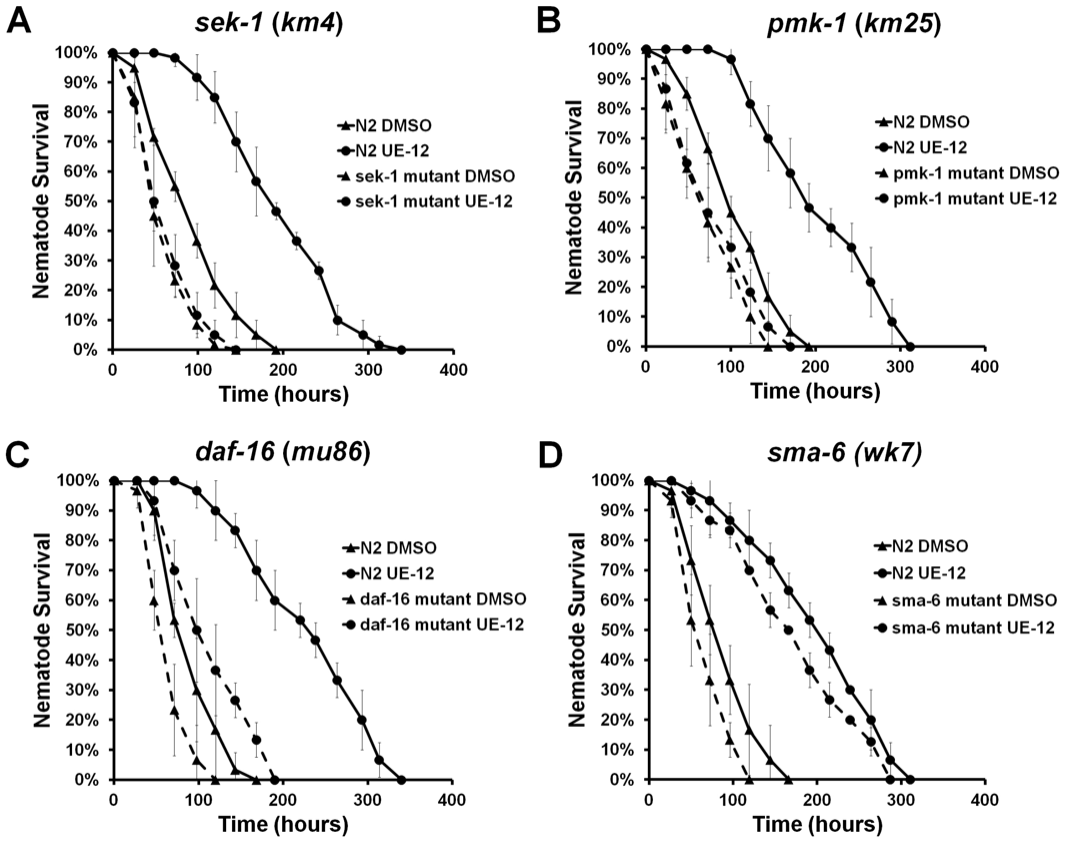
UE-12 requires the *C. elegans* p38 MAP kinase and insulin-like signaling pathways to confer its full protective effect. The survival curves of wild-type N2 and (A) *sek-1 (km4)* mutant animals, (B) *pmk-1 (km25)* mutants, (C) *daf-16 (mu86)* mutants and (D) *sma-6 (wk-7)* mutants following treatment with UE-12 or DMSO. The effect of UE-12 is diminished in *sek-1 (km4)* and *pmk-1 (km25)* mutants whilst partially attenuated in *daf-16 (mu86)* mutants. In *sma-6 (wk7)* mutants, UE-12 demonstrated a similar effect as in wild-type N2 animals (*p*<0.0001). Data at each time point are the average of six wells with each well containing 20 animals per well. Data are representative of three independent experiments.

Since we used the liquid based assay rather than the conventional agar-based assay, we first determined the sensitivity of these well-characterized *C. elegans* mutants to killing by *S. aureus* in a liquid-based assay. In agreement with reports using an agar-based assay, the *sek-1 (km4)* mutant is more susceptible to killing by *S. aureus* in the liquid killing assay (supplementary material Fig. S3A) (Sifri et al., 2003). Additionally, we found that the *pmk-1 (km25)*, *daf-16 (mu86)* and *sma-6 (wk7)* mutants were also hypersensitive to *S. aureus* infection under the same conditions (*p*<0.0001) (supplementary material Fig. S3B–D). The ability of UE-12 to enhance worm survival was completely abrogated in animals devoid of SEK-1 and PMK-1 (Fig. 4A,B, *p*>0.05) and partially attenuated in the *daf-16 (mu86)* null mutant (Fig. 4C, *p*<0.0001). In contrast, the protective effect of UE-12 was unaffected in the *sma-6 (wk7)* mutant infected by *S. aureus* (Fig. 4D, *p*<0.0001) even though the *sma-6* mutation conferred enhanced sensitivity to *S. aureus* (supplementary material Fig. S3D). This suggests that the SMA/TGF-β pathway is not required for the anti-infective activity of UE-12. On the other hand, failure of UE-12 to protect *sek-1 (km4)* and *pmk-1 (km25)* mutants indicates that the p38 MAP kinase signaling pathway is necessary for the anti-infective activity of UE-12. Although a significant lifespan extension was observed in the *daf-16 (mu86)* mutant infected by *S. aureus*, the effect was less pronounced than that observed in wild-type *C. elegans*, suggesting that the positive effect of UE-12 was only partially dependent on the insulin-like signaling pathway.

### UE-12 induces PMK-1 and DAF-16-regulated genes

To gain further insights into how p38 MAP kinase and insulin signaling mediate the protective effects of UE-12, we performed transcriptome analysis on a selected panel of PMK-1 and DAF-16-regulated genes using quantitative real-time PCR. The selected PMK-1-regulated genes comprised mainly presumptive antimicrobial candidates, namely CUB-like genes (C17H12.8, *dod-24*, F35E12.5 and F55G11.8) (Alper et al., 2007), ShK toxin genes (T24B8.5 and C14C6.5) (Nicholas and Hodgkin, 2004; O'Rourke et al., 2006) and C-type lectin genes (*clec-67* and M02F4.7) (Nicholas and Hodgkin, 2004; O'Rourke et al., 2006; Alper et al., 2007). These genes were also highly regulated in response to pathogen infection (Troemel et al., 2006). Total RNA was harvested from untreated or UE-12-treated *S. aureus*-infected nematodes at 48 hpi. At this time point, ∼20% mortality was observed in the untreated infected worms.

**Fig. 5.**
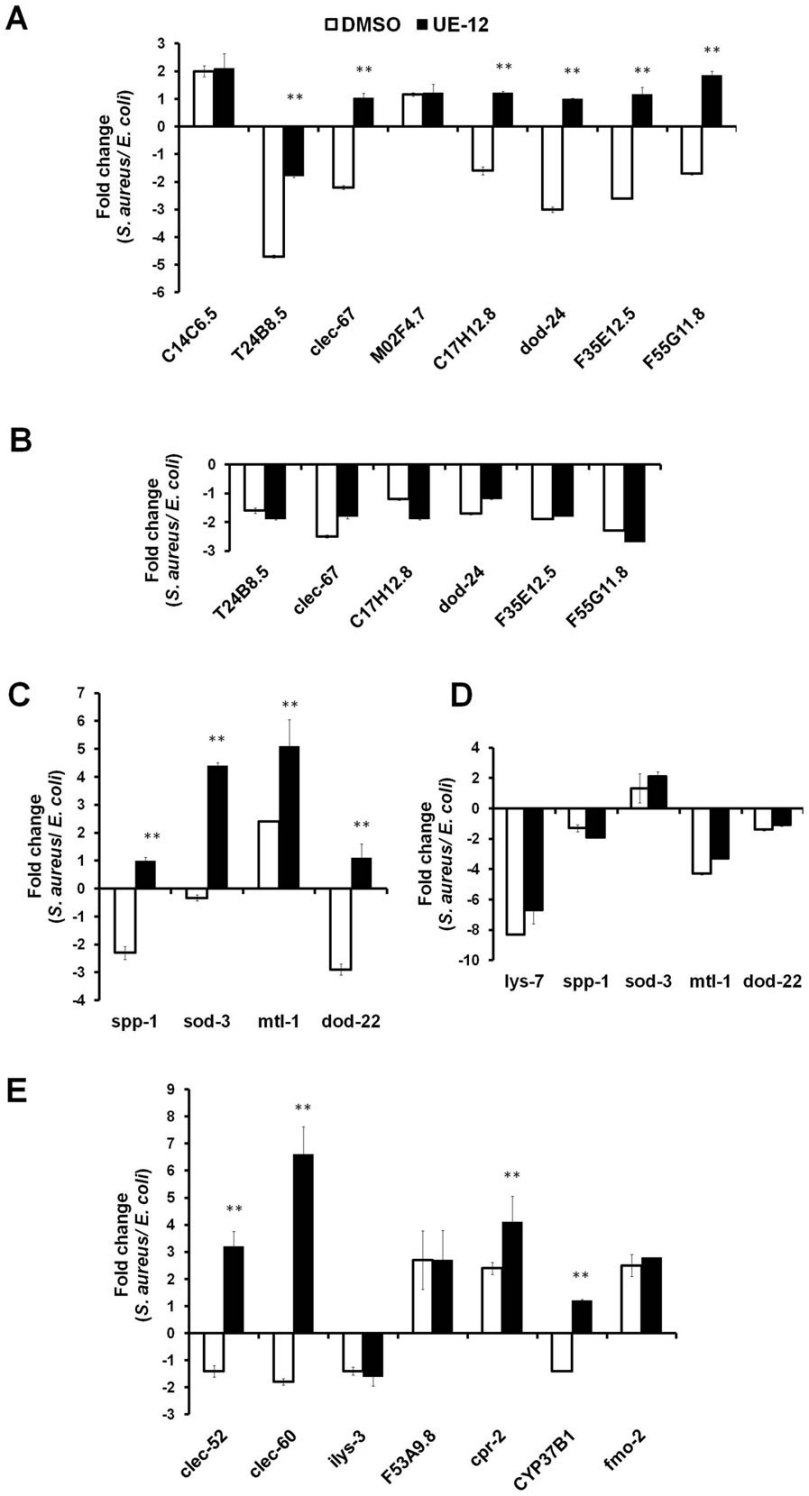
The expression of putative immune response genes is modulated in worms exposed to UE-12. (A,C,E) Selected panel of immune response genes analyzed by qRT-PCR in UE-12-treated nematodes and compared to untreated animals (DMSO) following infection by *S. aureus*. The fold change of all genes tested is relative to the uninfected population (exposed to *E. coli* OP50). (A) PMK-1 regulated genes. (C) DAF-16 targets. (E) Genes that were reported to be highly regulated in *S. aureus* infection. (B) Six PMK-1 target genes that were found to be significantly modulated in the presence of UE-12 were tested in the *pmk-1 (km25)* mutant. (D) Five DAF-16 target genes that were found to be significantly modulated upon exposure to UE-12 were tested in the *daf-16 (mu86)* mutant. Data are the average of three replicates, each normalized to changes in three primer pairs that were found to not vary with infection. The error bars represent the standard error of the mean. **A significant difference between untreated (DMSO) and UE-12-treated worms (*p*<0.05).

Six of the eight PMK-1-regulated genes were downregulated (∼2–5-fold) following 48 hours infection by *S. aureus* of worms that were not treated with UE-12 (Fig. 5A). On the other hand, in infected worms treated with 200 µg/mL UE-12, five of these 6 genes – *clec-67*, C17H12.8, *dod-24*, F35E12.5 and F55G11.8 – demonstrated a 2.8–4-fold induction in expression upon supplementation with UE-12 (*p*<0.05), implying that UE-12 promoted the host antimicrobial response. UE-12 also significantly reduced the suppression of T24B8.5 from ∼5-fold to ∼2-fold (*p*<0.05) (Fig. 5A).

We next seek to confirm that the effect of UE-12 is dependent on the presence of *pmk-1*. We infected *pmk-1 (km25)* mutant worms with the pathogen in the presence and absence of UE-12 and harvested the RNA for real time PCR analysis. We failed to observe any significant difference in the expression of these genes in both treated and untreated worms (Fig. 5B) further supporting the significant role played by the p38 MAP kinase signaling pathway in activating host defense responses in the presence of UE-12.

As the effect of UE-12 was partially attenuated in *daf-16 (mu86)* mutants, we analyzed the expression of four selected DAF-16-regulated genes to evaluate the involvement of the insulin-like signaling pathway in the UE-12 mediated protective effect. These four genes were candidate antimicrobial genes (*spp-1* and *dod-22*) (Murphy et al., 2003; Alper et al., 2007) and stress adaptation genes (*mtl-1* and *sod-3*) (Murphy et al., 2003). In the absence of UE-12, *spp-1* and *dod-22* were downregulated (2–3-fold), *mtl-1* was upregulated (∼2.5-fold) whilst *sod-3* was unaffected (Fig. 5C). In infected worms treated with UE-12, all four genes were significantly induced by 3.3–7.5-fold (*p*<0.05) (Fig. 5C), indicating that UE-12 was able to boost the expression of DAF-16-regulated genes. To confirm that the modulation of these genes require the presence of DAF-16, we profiled the expression of all four DAF-16 targets as well as *lys-7* (Fig. 1H) in the *daf-16* (*mu86*) mutant background. No significant difference was observed in the expression of these genes in both treated and untreated *daf-16* mutant animals (Fig. 5D). Hence, we confirm that the effect of UE-12 is *daf-16* dependent.

A previous study reported that the expression of both *lys-7* and *dod-22* were co-regulated by the *dbl-1*/TGF-β signaling pathway (Alper et al., 2007). Therefore, we also tested the expression of these genes in UE-12-treated and untreated *sma-6* (*wk7*) mutant worms infected by *S. aureus*. Similar to the profile observed in wild-type N2 worms, in a *sma-6* mutant background, the suppression of *lys-7* and *dod-22* by *S. aureus* was significantly induced in the presence of UE-12 extract (supplementary material Fig. S4), suggesting that the modulation of these two genes by UE-12 did not require the presence of *sma-6*.

In addition, we measured transcript levels of seven genes that were strongly induced during the early phase of infection by *S. aureus* (4, 8 and 12 hours) (Irazoqui et al., 2010). They consist of candidate antimicrobial genes (*clec-52*, *clec-60*, *ilys-3*, F53A9.8 and *cpr-2*) and detoxification genes (*cyp-37B1* and *fmo-2*). We found that during the late stage of infection (48 hpi), expression of *clec-52*, *clec-60*, *ilys-3* and *cyp-37B1* was downregulated, an effect that was reversed when infected worms were exposed to UE-12 (Fig. 5E). Specifically, the expression of *clec-52*, *clec-60* and *cyp-37B1* was induced significantly (*p*<0.05). Moreover, *cpr-2* demonstrated a further induction (*p*<0.05). At this late time point, the expression of *ilys-3*, F53A9.8 and *fmo-2* remained unchanged in treated and untreated animals (Fig. 5E). The fold difference for genes significantly modulated by UE-12 is shown in Table 1.

**Table 1.**
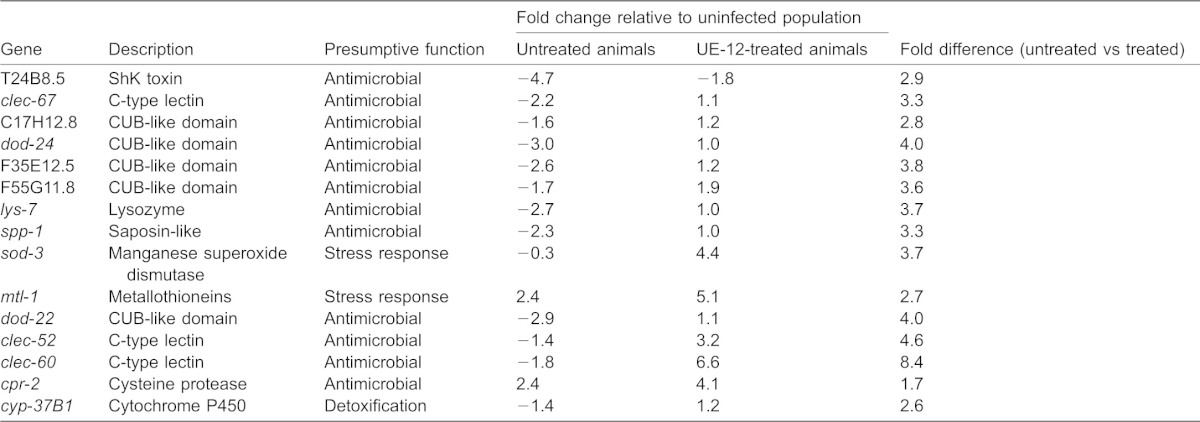
*C. elegans* genes significantly modulated by UE-12

We further tested if UE-12 is able to stimulate host innate immunity in the absence of infection. We measured mRNA levels of 11 immune effectors in worms grown in the presence and absence of UE-12 by qRT-PCR. Interestingly, the expression of none of these genes was significantly altered by UE-12 (supplementary material Fig. S5), implying that UE-12 does not affect the basal expression of these genes. Hence, we conclude that the effect of UE-12 is not a general stimulation of host immunity; instead, UE-12 modulates the host immune response towards pathogen infection.

In general, we noticed the suppression of host putative antimicrobial genes following 48 hours infection by *S. aureus*. Conversely, treatment with UE-12 resulted in a significant induction of 12/16 (75%) host antimicrobial genes tested. Thus, the protective effect of UE-12 is through the enhanced expression of host putative immune effectors upon pathogen infection, leading to the increased ability of infected nematodes to clear the bacteria and survive infection by *S. aureus*.

### Eupatorin is the active ingredient that contributes to the anti-infective effect of UE-12

**Fig. 6.**
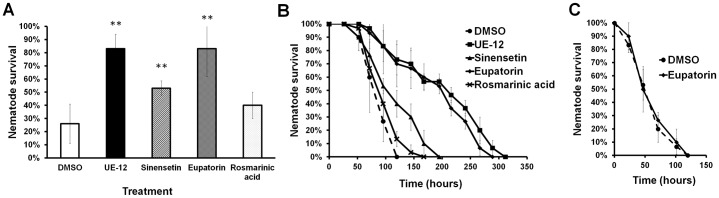
Eupatorin exerts a similar effect as UE-12 crude extract. (A) *S. aureus* infection assay of wild-type nematodes exposed to sinensetin, eupatorin and rosmarinic acid, respectively. The graph depicts the mean percentage of survival ± SD at 96 hpi of three replicates from a representative of two individual experiments. (B) Survival of *S. aureus*-infected worms is significantly improved over time upon supplementation with eupatorin. In a pair-wise comparison to untreated animals (DMSO) using log-rank tests, the difference is significant (*p*<0.0001). (C) The effect of eupatorin is completely impaired in *pmk-1 (km25)* mutants. The graph shows the mean ± SD of six replicates (20 nematodes/replicate) from a representative of three independent assays. **A significant difference between untreated (DMSO) and treated worms (*p*<0.05).

The availability of anecdotal evidence on the use of *O. stamineus* in folk medicine has led to substantial interest in *O. stamineus* and its traditional uses as indicated by the extensive volume of research devoted to this plant, including the identification of the plant extract phytochemical content. Several active chemical constituents that are prominently present in this plant are flavonoids (sinensetin and eupatorin) (Laavola et al., 2012; Yam et al., 2012) and a caffeic acid derivative (rosmarinic acid) (Olah et al., 2003; Pan et al., 2011). The chemical structures of sinensetin, eupatorin and rosmarinic acid are presented in supplementary material Fig. S6. We screened these compounds for their contribution to the anti-infective activity of UE-12. Both flavonoids sinensetin and eupatorin were able to enhance the survival of S. *aureus*-infected nematodes (Fig. 6A). At 96 hpi, a time point when the survival rate of untreated infected animals remained at 26%, sinensetin and eupatorin significantly increased survival to 53% and 83%, respectively (*p*<0.05) (Fig. 6A). Subsequently, we performed an extended time course based survival assay with a higher number of worms to validate these results and obtain the TD_mean_. From the time course study, we observed that eupatorin displayed equally effective curing activity as UE-12 whilst sinensetin weakly promoted nematode survival (Fig. 6B). The worms infected by *S. aureus* in the absence of any treatment had a TD_mean_ of 94.4±4.5 hours. In the presence of eupatorin, the TD_mean_ achieved 198.4±12.8 hours, which was comparable to that of UE-12 (203.5±14 hours) and significantly longer than that of untreated animals (*p*<0.0001). Sinensetin and rosmarinic acid only marginally extended the TD_mean_ to 117.2±8.2 hours and 100.5±5.2 hours, respectively (*p*>0.0001). Moreover, similar to the lack of anti-infective effects of UE-12 on *S. aureus*-infected worms (Fig. 4B), eupatorin failed to protect *pmk-1 (km25)* mutants from infection (Fig. 6C). Together, these findings suggest that eupatorin is likely the main bioactive component in UE-12 that promotes the survival of *S. aureus*-infected worms.

## DISCUSSION

Natural products have long been a source of therapeutics and continue to contribute significantly to the development of today's pharmaceuticals (Cragg et al., 1997). Previous studies on validation of traditional uses or on drug discovery have focused more on the antibacterial potential of medicinal plants (Cowan, 1999; Mahady et al., 2008). However, these bactericidal compounds are still capable of promoting the emergence of resistance. The ability of plant-derived natural products to boost the competence of the host immune system to defend against pathogen attack has received much less attention although this strategy may be just as important in combating infection. Taking advantage of the tractability and simplicity of *C. elegans* as an intact host model, we characterized the anti-infective effect of *O. stamineus* leaf extract (UE-12) in the context of a whole organism. As *O. stamineus* leaves are consumed daily by a number of different cultures and have good anecdotal evidence for treatment of infections, diabetes and hypertension (Ameer et al., 2012), the leaf extract is expected to not cause undesirable adverse effects. We provide evidence that the aqueous extract of *O. stamineus* leaves protect the host from infection by modulating the host innate immune response towards *S. aereus*. The extract modulated the host immune response via the evolutionarily conserved p38 MAP kinase pathway and insulin-like signaling pathway. Most notably, the expression of putative immune effectors regulated by PMK-1 and DAF-16 was induced in infected worms following exposure to this extract. We also present evidence that the immunomodulatory effect of this extract is most likely contributed by eupatorin, a flavonoid compound present in *O. stamineus* extract.

Studies to evaluate the antimicrobial properties of *O. stamineus* showed that the aqueous extract exhibited antibacterial action against *S. aureus* (Ho et al., 2010; Alshawsh et al., 2012). However, the MIC value reported was 1.56 mg/mL, which is much higher than the concentration used in our studies i.e. 200 µg/mL. Recently, Alshawsh et al. reported that *O. stamineus* extract has cellular immunomodulatory effects *in vitro* (Alshawsh et al., 2012). Specifically, *O. stamineus* significantly stimulated the proliferation of peripheral blood mononuclear cells in a dose-dependent manner, suggesting a role in modulating the cellular immune response. The protective role of this extract in our study, however, proposes a novel activity of targeting the innate immune response.

*C. elegans* has no adaptive immune system and the animal is believed to depend purely upon the secretion and action of antimicrobial molecules, including lysozymes (Schulenburg and Boehnisch, 2008), lectins (Schulenburg et al., 2008), caenopores (Roeder et al., 2010) and antibacterial factors (Kato et al., 2002). The primary route and major site of infection for *S. aureus* in a *C. elegans* model is through the intestine (Sifri et al., 2003). *S. aureus* accumulate and distend the worm intestinal lumen thereby killing the host. Interestingly, most of the antimicrobial effector genes modulated by UE-12 (*clec-67*, *dod-24*, *lys-7*, *spp-1*, *dod-22*, *clec-52* and *clec-60*) are expressed predominantly in the intestine. Hence, the induced expression of these genes aid in the elimination of the bacteria from the host, eventually clearing the infection and resulting in increased survival of worms with low numbers of intestinal live *S. aureus*. A previous study has reported that *lys-7* mRNA levels are upregulated at 24 hpi, followed by a decrease at 36 and 48 hpi by *S. aureus* strain ATCC11632 based on a liquid assay (JebaMercy et al., 2011). Nevertheless, in our study, both GFP and real time PCR data showed a homogenous suppression of *lys-7* expression at 24 and 48 hpi (Fig. 1G,H). This difference could be explained by the different experimental settings such as the virulence of bacterial strain tested, bacterial culture conditions and assay medium as previously reported for other human pathogens (Tan et al., 1999; Garsin et al., 2001; Lee et al., 2011).

As the inexorable invasion by antibiotic-resistant pathogens continues to threaten the community, there is a pressing need for novel forms of therapy. Anti-infectives that target bacterial virulence or host immunity without affecting microbial cell viability offer an alternative to conventional antibiotics. Recently, through high throughput virtual screening, Khodaverdian et al. discovered a small number of compounds that inhibited the production of hemolysin and phenol-soluble modulin of MRSA without inhibiting bacterial growth (Khodaverdian et al., 2013). Interference with bacterial quorum sensing and biofilm production also appears to represent a promising intervention strategy (Wu et al., 2004; Balaban et al., 2007). At this juncture, we cannot rule out the possibility that UE-12 extract may also affect bacterial virulence as the plant extract contains numerous active constituents (Yam et al., 2012). It has been reported that plants possess compounds that can attenuate the virulence of a pathogen by inhibiting virulence factors including quorum sensing and biofilm formation (Adonizio et al., 2008; Rudrappa and Bais, 2008). The effect of UE-12 extract on *S. aureus* protease, hemolysin, lipase and biofilm production were accessed through several simple *in vitro* approaches but we did not observe any significant inhibition in production of these factors (data not shown), indicating that the extract did not disrupt the production of these virulence factors. However, we have not ruled out the possibility that these factors may be inactivated upon exposure to UE-12. The effect of UE-12 on other bacterial virulence traits is yet to be determined.

Immunomodulatory drugs targeting host immunity have been proposed as a possible alternative to conventional antibiotics (Hancock et al., 2012). This approach has been minimally employed to combat bacterial infection but is a mainstay of anti-cancer (Waldmann, 2006) and anti-viral therapy (DesJardin and Snydman, 1998; Trnková et al., 2012). A recent study by Kindrachuk et al. demonstrated that the secreted peptide from bacteria, bacteriocin, was able to augment chemokine production by human cells, leading to protection against bacterial infection in animal models (Kindrachuk et al., 2013). In the *C. elegans* model, a small molecule RPW-24 (Pukkila-Worley et al., 2012) and the alkaloid compound Harmane (Jakobsen et al., 2013) were reported to be able to modulate or stimulate the host innate immune response upon pathogen infection, resulting in enhanced survival of infected nematodes. Additionally, the red seaweed *Chondrus crispus* has recently been reported to promote the host immune responses towards *Pseudomonas aeruginosa* infection by inducing the expression of *C. elegans* innate immune genes via highly conserved pathways (Liu et al., 2013). We have shown that UE-12 activity is dependent primarily on the p38 MAP kinase and secondarily on the insulin/DAF-2 signaling pathways. As these signaling pathways are highly conserved evolutionarily, it is conceivable that findings from the nematode model could be extended to higher organisms, including humans. The immunomodulatory effect observed in this study, together with the findings in aforementioned studies, provide a scaffold upon which further approaches to treating bacterial infections by targeting the immune system are possible.

Hyperactivation or dysregulation of the immune response may contribute to life-threatening infectious and inflammatory disorders. Therefore, it is vital to study the toxic effect of a compound that confers any survival advantage to the host. The *C. elegans* whole animal model allows for early and direct assessment of *in vivo* drug efficacy, thus, eliminating compounds that are toxic to the host or with poor pharmacokinetic properties. Unlike the previously reported small molecule RPW-24 that exerted an immunostimulatory effect on the host (Pukkila-Worley et al., 2012), no observable toxic effect was detected on adult worms as well as the offspring exposed to UE-12. Furthermore, the extract did not affect the basal expression of the immune response genes under uninfected conditions. In light of this, we assume that UE-12 did not over stimulate host immunity, but rather, modulated the immune response at a level sufficient to overcome the infection whilst having no detrimental effect on the host. Thus, our findings encourage the development of the largely unexplored natural resources for immunomodulatory properties as alternative forms of treatment.

In summary, through a series of *in vivo* experiments involving infection of an intact host, we provide evidence that the aqueous extract of *O. stamineus* leaves markedly promotes the survival of *S. aureus*-infected nematodes by modulating the expression of immune-related genes. Of note are the candidate antimicrobial genes which are crucial for host defense during infection and furthermore, the extract did not have any notable adverse effect on the animal or its offspring. We further suggest that the flavanoid eupatorin is likely to be the active element in this extract that confers a protective advantage towards pathogen infection.

## MATERIALS AND METHODS

### Bacterial and nematode strains and growth conditions

*S. aureus* strain NCTC8325-4 was routinely propagated on Trypticase Soy (TS) agar (Pronadisa, Spain) at 37°C whilst *E. coli* strain OP50 was cultured on Luria Bertani (LB) media (Pronadisa, Spain) supplemented with streptomycin (100 µg/mL). The *C. elegans* strains used in this study were: wild-type N2 Bristol (Brenner, 1974), *pmk-1* (*km25*) (Troemel et al., 2006), *sek-1* (*km4*) (Troemel et al., 2006), *daf-16* (*mu86*) (Evans et al., 2008), *sma-6* (*wk7*) (Luo et al., 2009) and transgenic strain p*lys-7::GFP* (Evans et al., 2008). All worm strains were obtained from the Tan Laboratory, Stanford University, USA. Growth and manipulation of *C. elegans* were performed as previously described (Brenner, 1974).

### Preparation of extract and compounds

The preparation of UE-12 aqueous extract has been previously described (Kong et al., 2014). Briefly, *Orthosiphon stamineus* leaves were collected and taken through a series of stringent microbial tests as well as Atomic Absorption Spectrophotometry (AAS) to test for heavy metal presence. The leaves were then dried, ground into fine powder which was used in a water-based extraction. The microbial and trace metal element tests were repeated again on the extraction yield. The product was examined for the presence of functional groups/molecules by Fourier transform infrared spectroscopy (FTIR) and High-performance liquid chromatography (HPLC). Sinensetin (CAS no. 2306-27-6), eupatorin (CAS no. 855-96-9) and rosmarinic acid (CAS no. 20283-92-5) were purchased from ChromaDex®, Inc. (Irvine, CA). The extract and compounds were dissolved in dimethyl sulfoxide (DMSO) and filtered through a 0.2 µm membrane filter (Sartorius Stedim, Germany) followed by storage at −20°C in a desiccated storage container.

### Growing worms for infection and other assays

To eliminate the confounding effects of progeny during the scoring of surviving worms, wild-type and mutant *C. elegans* were treated to RNAi knockdown of the *pos-1* gene which resulted in worms laying unhatched eggs (Tabara et al., 1999). The dsRNA directed against *pos-1* was introduced into the worms by feeding. Briefly, the *pos-1* RNAi clone was cultured in 100 mL LB medium supplemented with 100 µg/mL carbenicillin and incubated overnight at 37°C. The culture was concentrated 25-fold before seeding onto Nematode Growth (NG) agar supplemented with 1 mM Isopropyl β-D-1-thiogalactopyranoside (IPTG) (Promega, USA) and 100 µg/mL carbenicillin and incubated at room temperature for 24 hours. Following synchronization by hypochlorite treatment, the eggs of wild-type N2 and mutants were plated onto *pos-1* RNAi plates and allowed to grow for 45 hours at 25°C until they reached young adult stage and were ready to be used in the experiment.

### Infection assay

Infection assays were performed as previously described (Kong et al., 2014). Worm M9 buffer and the overnight culture of *S. aureus* in TS broth with 10 µg/mL of cholesterol at a ratio of 4:1 (v/v) was dispensed into a 24-well plate prior to transferring wild-type N2 worms. In the treatment wells, UE-12 aqueous extract was added at 200 µg/mL or indicated concentrations for dose-dependent tests. To investigate the effect of UE-12 in mutant background worms, the assay was prepared as above except that wild-type N2 nematodes were replaced by the mutant strain of interest. To determine which bioactive compound in UE-12 promoted the survival of nematodes infected with *S. aureus*, UE-12 was substituted with 50 µM of sinensetin, eupatorin or rosmarinic acid. In control wells, the extract was replaced with 1% DMSO or *S. aureus* was replaced by *E. coli* OP50. Twenty nematodes were transferred into each well and the plate was incubated at 25°C. The total number of worms amounted to 120 in six wells representing six technical replicates. Nematode survival was monitored and scored manually every 24 hours. At least three independent experiments were performed.

### Lifespan assay

The *C. elegans* lifespan assay was carried out in liquid medium in a 24-well plate (Yu et al., 2010). A single colony of *E. coli* OP50 was inoculated in 100 mL of LB broth supplemented with 500 µg/mL streptomycin and incubated overnight at 37°C. The culture was concentrated 25-fold before M9 buffer was added. UE-12 at 200 µg/mL was added into the treatment wells whilst 1% DMSO was added into the control wells. Age-synchronized young adult nematodes were transferred manually to the medium and plates were incubated at 25°C. Animals were scored daily as alive or dead by gentle prodding with a platinum wire. To eliminate any possible effect of UE-12 on *E. coli* OP50 that might in turn extend or shorten the *C. elegans* lifespan, a lifespan assay was conducted with heat-killed *E. coli* OP50. The concentrated culture was heat-killed at 65°C for 30 mins (Van Voorhies et al., 2005) before adding the bacteria into the liquid medium. Following heat treatment, no live cells could be detected by plating undiluted cultures on LB agar. The experiment was performed in duplicate.

### Reproduction assay

The effect of UE-12 on *C. elegans* reproduction was assayed by picking individual L4 wild-type hermaphrodites onto NG agar supplemented with 200 µg/mL of UE-12. To provide better exposure towards the extract, the UE-12 extract was spread on the NG agar before the seeding of heat-killed *E. coli* OP50. The control plates were supplemented with 1% DMSO. Thereafter, gravid adult worms were moved to a fresh plate each day until reproduction ceased. The offspring on the plates were allowed to grow at 25°C and the number of progeny at the L2 and L3 stage was determined. The total number of progeny from the various plates was enumerated as the final brood size. This experiment was independently repeated twice.

### Feeding rate assay

The experiment was conducted as that described previously (Dharmalingam et al., 2012) with minor modifications. Worms were prepared and exposed to *S. aureus* according to the infection assay protocol in the presence and absence of 200 µg/mL UE-12. At 2, 4, 8 and 10 hpi, the pharyngeal pumping rate of 5 individual worms was counted for 5 seconds under a microscope (Leica Microsystem M205 FA, Germany). A pharyngeal pump was strictly defined as a complete backward movement of the terminal bulb grinder (Keane and Avery, 2003). The average number of pharyngeal pumps in 5 worms (pumps/second) was calculated and used for statistical analysis. The experiment was repeated twice.

### Colony forming units (CFU) assay

*In vivo* enumeration of bacterial CFU within the *C. elegans* gut was performed as previously described (Ooi et al., 2012) with minor modifications. Briefly, the worms were infected by *S. aureus* in the presence and absence of UE-12 as described above. After 4, 12, 24, 48 and 72 hours, 10–12 worms were washed with 10 µg/mL gentamicin in 25 mM Levamisole (Lev) (Sigma–Aldrich, USA) to remove bacteria present on the worm cuticle. The worms were then homogenized using a motorized pestle and lysates were serially diluted and plated on TS agar. After an overnight incubation at 37°C, colonies were counted and the CFU counts per worm were determined. To establish growth of *S. aureus* in liquid medium in the presence of UE-12, the liquid medium, comprising 20% *S. aureus* culture and 80% M9 buffer, was prepared as described above. At the indicated time points, 10 µL culture was removed from the medium, serially diluted and plated on TS agar. Three independent experiments were performed.

### Visualization and scoring of transgenic *C. elegans* p*lys-7*::GFP

The transgenic *C. elegans* strain carrying a *lys-7* promoter fused to the green fluorescent protein open reading frame (ORF) was used to visualize the expression of *lys-7*. p*lys-7*::GFP transgenic worms were subjected to *S. aureus* infection in the presence and absence of UE-12 as described in the infection assay above. A total of 20 live transgenic worms were picked and paralyzed with 5 mM Levamisole at 24 and 48 hpi. The worms were mounted on a 2% agarose gel pad for observation at 100× magnification. Fluorescence intensity was observed under a Leica DM5000B automated upright fluorescent microscope equipped with an I3 long-pass GFP filter. Fluorescence micrographs were collected using a ProgRes C10 Plus digital microscope camera (Jenoptic Laser, Jena, Germany) and Leica Application Suite software (Leica Microsystems, Wetzlar, Germany). Each image series was captured on identical settings. Each worm was given a score of 1, 2 or 3 as shown in Fig. 1D–F, indicating low, medium and high fluorescence intensity, respectively. Observations were made on transgenic worms grown on *E. coli* OP50 (uninfected) and on worms infected with *S. aureus* in the presence and absence of extract. At least three independent replicates were performed.

### Total RNA isolation and quantitative RT-PCR (qRT-PCR) analysis

Animals were treated essentially as described above according to the specific experimental conditions, after which, total RNA was extracted from a synchronized population of worms (∼2500 worms) using TRIzol reagent (Invitrogen, USA) according to the manufacturer's instructions, followed by purification on RNeasy columns (Qiagen, Germany). qRT-PCR reactions were hereafter performed with DNase-treated total RNA using the iScript One-Step RT-PCR kit with SYBR green detection according to the manufacturer's instructions (BioRad Laboratories, USA) on the Bio-Rad CFX96 Touch Real-Time PCR Detection System. The system was used for amplification and quantification of the products. Specificity of amplification was confirmed by melt curve analysis after amplification. Primers for qRT-PCR were based on those previously published (Troemel et al., 2006; Alper et al., 2007; Irazoqui et al., 2008). Normalized threshold cycle values were used to calculate fold increase or decrease of RNA levels in samples from test animals as compared to controls. The C_t_ values were normalized to changes in three primer pairs (*ama-1*, pan-actin (act-1, 3, 4) and F44B9.5) that were found to not vary with infection.

### Statistical analysis

For all experiments, six replicates (in six wells) per trial were carried out (with a total of 120 worms) for statistical purposes. Nematodes were classified as dead when they failed to respond to touch and no pharyngeal pumping was observed. Worms that died because of bursting vulva were censored from further analysis. Differences in survival and lifespan of *C. elegans* between treatment (UE-12 or its bioactive compounds) group and control group (DMSO) in both wild-type and mutant animals were assessed by the Log-rank (Mantel–Cox) significance test using *StatView* version 5.0.1 (SAS Institute, Inc.). The ordinal data for quantitative GFP experiments were analyzed with the Mann–Whitney U-test. For other assays, data were analyzed and compared by using the unpaired, two-tailed Student's *t*-test. Fold changes in the qRT-PCR analysis were also compared using Student's *t*-tests.

## Supplementary Material

Supplementary Material
