# Supplementary material for: Orthosiphon stamineus protects Caenorhabditis elegans against Staphylococcus aureus infection through immunomodulation
Source: Biol Open. 2014 Jun 27;3(7):644–55. doi: 10.1242/bio.20148334 (PMC4154301; doi:10.1242/bio.20148334)
Supplement: Supplementary Material [file supp_3_7_644__index.html]

Orthosiphon stamineus protects Caenorhabditis elegans against Staphylococcus aureus infection through immunomodulation — Supplementary Material 

# *Orthosiphon stamineus* protects *Caenorhabditis elegans* against *Staphylococcus aureus* infection through immunomodulation

## bio.20148334 Supplementary Material

**Files in this Data Supplement:**

- Supplementary Material - Cin Kong et al. doi: 10.1242/bio.20148334
