# Supplementary material for: Orthosiphon stamineus protects Caenorhabditis elegans against Staphylococcus aureus infection through immunomodulation
Source: Biol Open. 2014 Jun 27;3(7):644–55. doi: 10.1242/bio.20148334 (PMC4154301; doi:10.1242/bio.20148334)
Supplement: Supplementary Material [file supp_bio.20148334_bio.20148334-s1.pdf]

Supplementary Material
Cin Kong et al. doi: 10.1242/bio.20148334

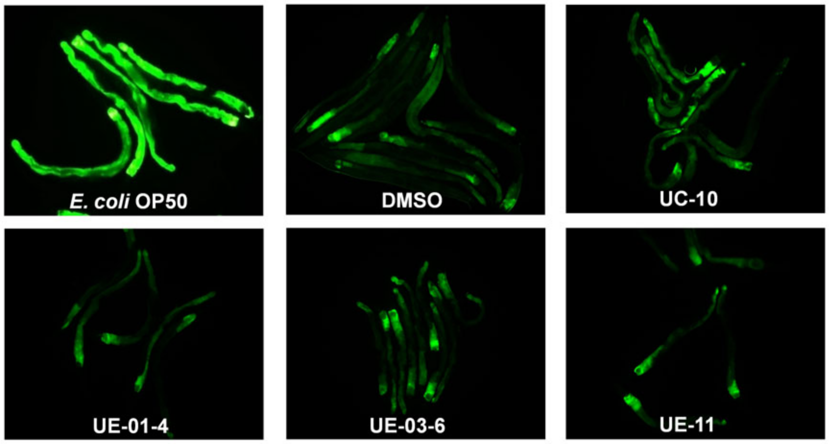

UC-10 : Compound UC-10
UE-01-4 : *Nypa fruticans* root butanol extract
UE-03-6 : *Swietenia macrophylla* seed butanol extract
UE-11 : *Eurycoma longifolia* root water extract

Fig. S1. Fluorescence micrographs of p*lys-7::gfp* transgenic *C. elegans* (100× magnification). Worms showed fluorescence expression when cultured under normal conditions with *E. coli* OP50 as the food source. Decreased GFP intensity was observed when the transgenic worms were infected with *S. aureus* for 48 hours. Treatment with UC-10, UE-01-4, UE-03-6 and UE-11 were unable to restore the repressed GFP intensity.

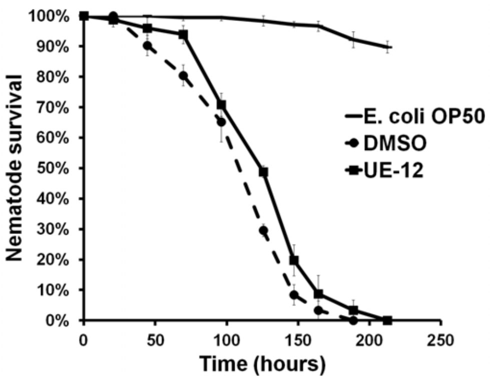

Fig. S2. The survival curves of *S. aureus*-infected *C. elegans* upon exposure to UE-12 extract on the conventional agar-based assay. Enhanced survival of infected worms can be seen in the presence of UE-12 but the effect is weak if compared to that seen in the liquid-based assay. Results are expressed as mean ± SD from a representative of at least two individual assays.

Biology Open

Biology Open (2014) 000, 1–12 doi:10.1242/bio.20148334

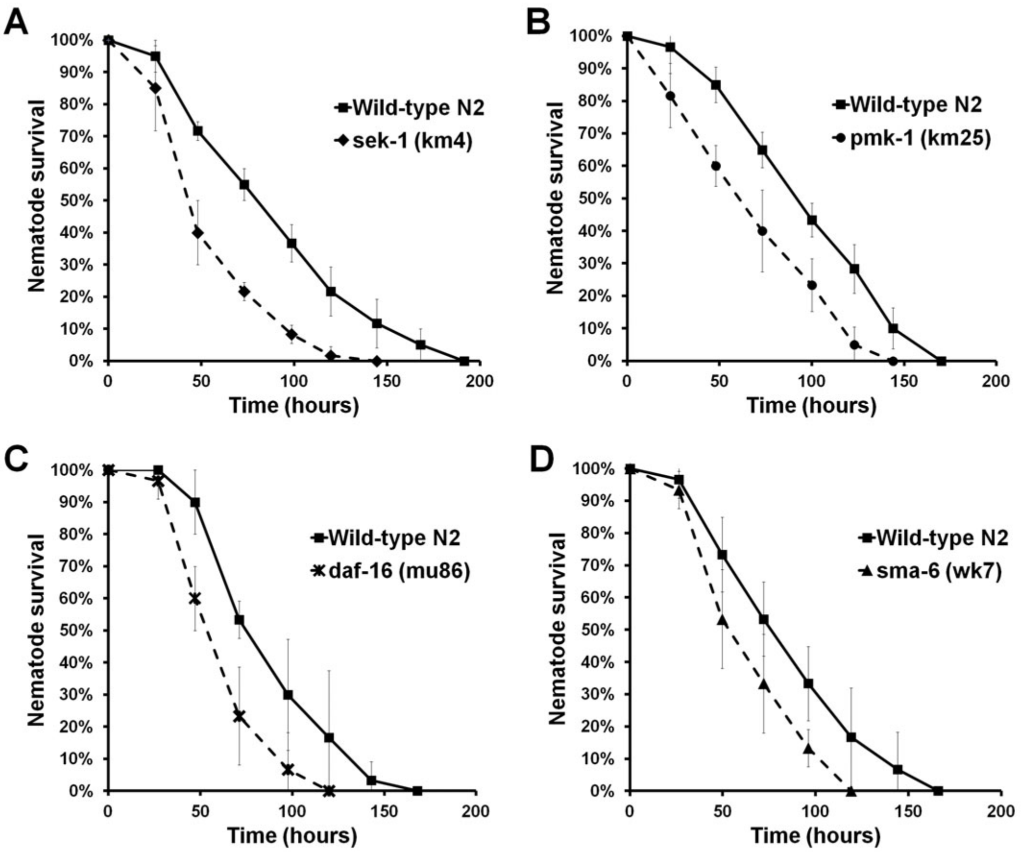

Fig. S3. *C. elegans* mutants with enhanced susceptibility to *S. aureus*. Survival curve of wild-type N2 *C. elegans* (square) and (A) *sek-1 (km4)* (diamond), (B) *pmk-1 (km25)* (circle), (C) *daf-16 (mu86)* (cross) and (D) *sma-6 (wk7)* (triangle) mutants fed on *S. aureus*. The graph presents the mean ± SD of six replicates (20 nematodes/replicate) from a representative of two independent assays.

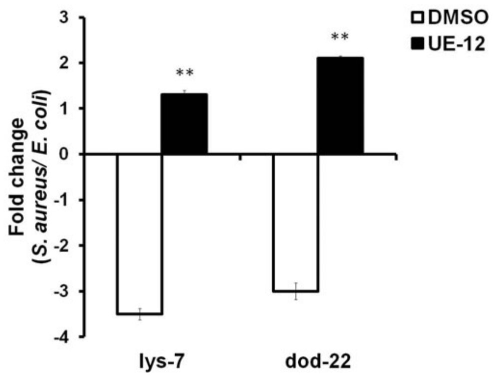

Fig. S4. The expression of *lys-7* and *dod-22* was measured in the *sma-6 (wk7)* mutant. Data are the average of two replicates, each normalized to changes in three primer pairs that were found to not vary with infection. The error bars represent the standard error of the mean. **A significant difference between untreated (DMSO) and UE-12-treated worms ($p<0.05$).

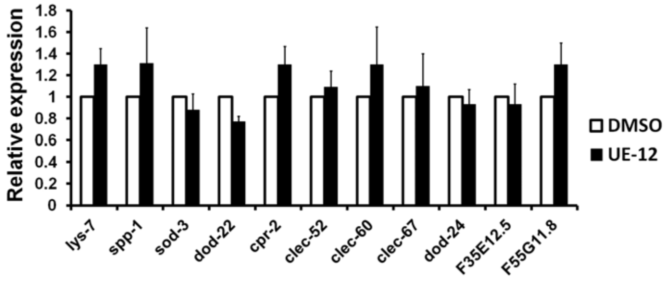

Fig. S5. Effect of UE-12 on selected *C. elegans* immune response genes under uninfected conditions. *C. elegans* were exposed to the normal food source *E. coli* OP50 and mRNA levels of the 11 genes were measured by qRT-PCR. None of these genes show significant modulation in the presence of UE-12. Columns represent mean ± SEM of two biological replicates, each normalized to three housekeeping genes.

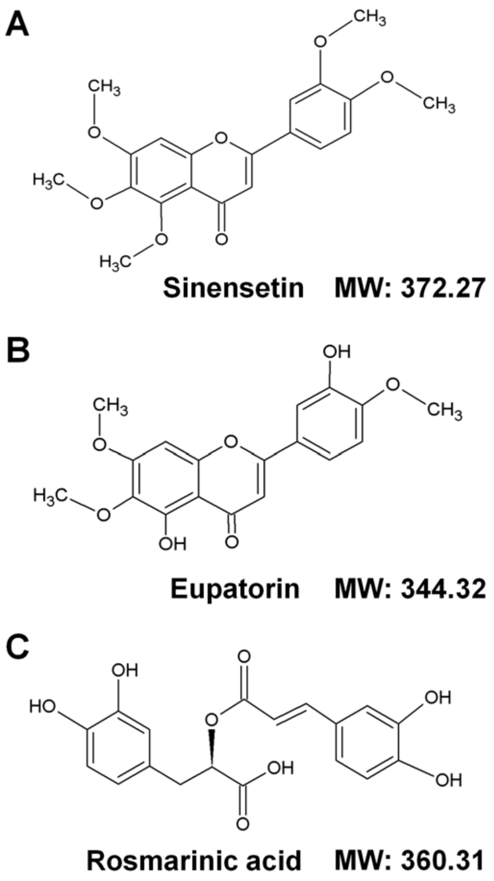

Fig. S6. The chemical structure of known active compounds in *Orthosiphon stamineus* extract. The structures of (A) sinensetin ($C_{20}H_{20}O_7$), (B) eupatorin ($C_{18}H_{16}O_7$) and (C) rosmarinic acid ($C_{18}H_{16}O_8$) are shown along with the respective molecular weight (MW).
